# Efferocytosis: The Silent Guardian of Tissue Homeostasis and Cardiovascular Health

**DOI:** 10.3390/jcdd13010021

**Published:** 2025-12-31

**Authors:** Wenting Peng, Yuhao Song, Shengxi Gu, Ye Zhu, Ying Li

**Affiliations:** 1 Department of Cardiology, West China Hospital, Sichuan University, Chengdu 610041, China or 2023224025271@stu.scu.edu.cn (W.P.); songrainhowe@163.com (Y.S.); 15182585147@163.com (S.G.); 2 Department of Cardiology, West China School of Public Health and West China Fourth Hospital, Sichuan University, Chengdu 610041, China

**Keywords:** efferocytosis, cardiovascular diseases, atherosclerosis, apoptotic cells, necrosis

## Abstract

Rapid and effective clearance of apoptotic cells, known as efferocytosis, is essential for maintaining tissue homeostasis. Efferocytosis removes apoptotic cells before the occurrence of membrane rupture from which the cell contents, often inflammatory and toxic, are released into surrounding tissues. Through this way, efferocytosis protects the surrounding tissues from toxic enzymes and oxides inside the apoptotic cells as well as from cellular contents such as anti-proteinase and cystatins. Driven by the ongoing advancements in bioinformatics and molecular biology, many researchers have explored the mechanism of efferocytosis and its association with systemic diseases. Multiple studies have demonstrated that impaired efferocytosis mechanisms significantly contribute to the onset and progression of chronic inflammation. The presence of chronic inflammation significantly exacerbates the advancement of cardiovascular diseases, including atherosclerosis, myocardial infarction, heart failure subsequent to myocardial infarction, and even myocarditis. This review aims to provide a brief introduction to the mechanisms involved in cellular efferocytosis, followed by an examination of the molecular and pathway aspects of efferocytosis with the risk of cardiovascular diseases, contributing to the identification of potential therapeutic targets for related diseases.

## 1. Introduction

Efferocytosis has become a topic of research interest in recent years. Based on genetics, biochemistry, and imaging, it has been revealed that efferocytosis acts differently from the classical form of phagocytosis [1]. During efferocytosis, phagocytosing cells engulf apoptotic cells and produce large vesicles containing apoptotic cells, called efferocytosis bodies. The production of anti-inflammatory cytokines and inhibition of pro-inflammatory cytokines occur during the process, suggesting that efferocytosis differs mechanistically from phagocytosis of pathogens and other opsonized particles, as it rather occurs in a state of immune quiescence [2].

A variety of cells, generally classified into two categories, namely professional phagocytes and nonprofessional phagocytes, can perform efferocytosis. Professional phagocytes include macrophages and immature dendritic cells, which perform efferocytosis at a higher rate and capacity [3]. Nonprofessional phagocytes include epithelial cells, endothelial cells, and fibroblasts. The stages of the removal of apoptotic particles remain the same for both professional and nonprofessional cells, and tissue-resident macrophages in the plasma and lungs are the main cell types involved in efferocytosis [4].

Apoptotic cells have a large number of ligands on their surface to ensure rapid phagocytosis and clearance, sometimes referred to as apoptotic cell–associated molecular patterns or ACAMPs. ACAMPs include modified carbohydrates, externalized proteins such as calmodulin, and, most typically, externalized negatively charged glycerophospholipid phosphatidylserine (PS), which is normally confined to the intracytoplasmic space but is externalized during apoptosis [5]. Efferocytosis can produce a large number of biokines, such as vascular endothelial growth factor and hepatocyte growth factor, which may promote renewal of apoptotic cells by the body. Efferocytosis also induces the production of anti-inflammatory cytokines and survival factors by macrophages.

## 2. Mechanism of Efferocytosis

**Figure 1:**
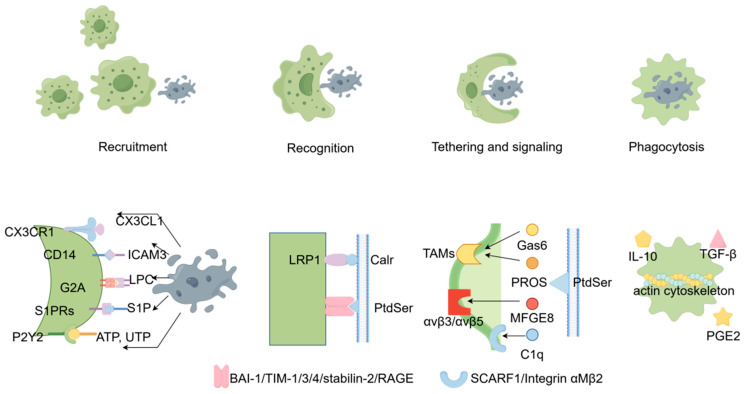
The process of efferocytosis. The process of efferocytosis involves four stages, namely recruitment, recognition, tethering and signaling, and phagocytosis. In the first stage, apoptotic cells (ACs) rapidly recruit efferocytic immune cells by releasing lipids (such as nucleotides [adenosine triphosphate and uridine triphosphate, ATP and UTP], lysophosphatidylcholine [LPC], S1P, and chemokines [such as CX3CL1]). Then, efferocytes recognize ACs via cell-membrane receptors that either directly bind ligands on the AC membrane or bind bridging molecules interacting with the AC membrane. In the final stage, phagocytes digest and degrade ACs, releasing post-efferocytosis signaling molecules.

Many cells are involved in efferocytosis, and the specific process can be divided into four parts, namely recruitment, recognition, tethering and signaling, and phagocytosis [6] (some researchers include tethering and signaling within recognition). The signaling network system of efferocytosis action is mainly composed of “find me”, “keep out”, “eat me”, and “do not eat me” signals [7], and the four steps of the process are achieved through the mutual recognition and action of different signals (shown in Figure 1).

### 2.1. Recruitment Phase

At the onset of apoptosis, apoptotic cells emit “find me” signals, which are sensed by motile phagocytes and yield to their recruitment. The signals include CX3CL chemokine ligand-1 (CX3CL1), intracellular adhesion molecule 3 (ICAM3), lysophosphatidylcholine (LPC), sphingosine-1-phosphate (S1P), nucleotides ATP and UTP, and other molecules [1]. These signals are transmitted via the extracellular fluid or peripheral blood to efferocytosis cells and are recognized by recognition receptors on the surface of these cells (G2A family of G-protein-coupled receptors (G2A), sphingosine-1-phosphate receptor (S1P) phosphate receptor (S1PR), the chemokine receptor CX3CR1, and the P2Y family of G-protein–coupled receptors (P2Y)) [8], directing phagocytes to the tissue region where apoptotic cells are located [9].

### 2.2. Recognition Phase

This phase involves binding between macrophages and apoptotic cells through binding ligands (“eat me” signals) on the surface of the apoptotic cells and receptors (“eat me” markers) on the surface of macrophages. Common “eat me” signals include PS, calreticulin (Calr), and existing molecules modified on the surface of apoptotic cells, but PS is the most important “eat me” signaling molecule [10,11]. PS can bind directly to phagocyte surface receptors, including the T-cell immunoglobulin mucin family (TIM) members TIM-1 and TIM-4, as well as BAI-1, stabilin-2, and the receptor for advanced glycation end products (RAGE) [12].

### 2.3. Tethering and Signaling Phase

In addition to binding directly to the receptors on the phagocyte surface, PS can also be bound by bridging molecules. Bridging molecules that mediate the recognition of PS are members of the tyrosine kinase receptor family (TYRO3, AXL, MerTK, TAM) on the surface of the phagocyte membrane. During this period, nonapoptotic cells send “do not eat me” signals, such as integrin-associated protein CD47 (one of the integrin-associated proteins, IAPs), whose balanced activity is essential for efficient efferocytosis [13], triggering the SIRPa receptor to resist efferocytosis and preventing the clearance of living cells [14]. PS can also act as a bridging molecule through the use of milkfat globule–epidermal growth factor 8 (MFGE8) or the complement component C1q. MFGE8, for example, connects PS on the surface of apoptotic cells on the one hand and αvβ3/αvβ5 integrins on the other hand, thereby acting as a bridge between the apoptotic cells and macrophages and facilitating the tethering step. The DEL-1 molecule bridging between PS on apoptotic cells and avb3 integrin on macrophages mediates the cytosolic burial of apoptotic cells by macrophages [15]. A newly identified bridging molecule, Annexin 1, promotes recognition and efferocytosis of apoptotic cells [16]; it is an intracellular protein in its normal state, but it is translocated to the outer surface of the cell membrane containing PtdSer patches during apoptosis.

### 2.4. Phagocytosis Phase

Efferocytosis phagocytes signal the apoptotic cells to be phagocytized by the p130cas–CrkII–Dock180 complex, activate Rac1, rearrange the actin cytoskeleton, and form phagosomes to complete the phagocytosis of the apoptotic cells [17,18]. The phagocytosis process is followed by the production of regulatory and anti-inflammatory cytokines, such as interleukin (IL)-10, transforming growth factor (TGF-β), and prostaglandin E2 (PGE2) [19].

The apoptotic process and the status of various signaling molecules are closely linked to the efferocytosis process (shown in Table 1). In recent years, an increasing number of studies have explored the effects of certain drugs or chemical components on the regulation of genes or signaling molecules in the efferocytosis process. Maimon et al. [20] reported that sept4/ARTS−/− mice exhibited impaired inflammatory regression, characterized by reduced apoptosis in polymorphonuclear (PMN) cells, which led to diminished efferocytosis and prevented macrophage recoding to pro-lysis macrophages to produce anti-inflammatory cytokines. This suggests that the blocked apoptotic process leads to a failure of efferocytosis and thus impaired inflammatory regression. In contrast, in recent years, a growing number of studies have investigated the regulation of targets in the efferocytosis process by drugs or chemical components in order to explore the feasibility of relevant drugs or chemicals for the treatment of efferocytosis-related diseases. Schutters et al. [21] found that RGD–anxA5 (Arg–Gly–Asp–AnnexinA5) enhanced phagocytosis of apoptotic cells by human acute monocytic leukemia cell line cells in vitro and in vivo. Targeting PS expressed on the cell surface is an attractive strategy for the treatment of inflammatory diseases; therefore, rationally designed RGD–anxA5 is a promising therapeutic agent. According to Inoue et al. [15], caspastatin promotes not only phagocytosis but also the binding of apoptotic cells to alveolar macrophages (AMs) to facilitate the clearance of apoptotic cells from airways, which can be used to promote efferocytosis effects. In humans, efferocytosis is necessary because if apoptotic cells are not cleared by efferocytosis, they undergo secondary necrosis and inflammatory cell contents are released into the extracellular space, which promotes autoimmunity and ongoing inflammation.

## 3. The Role of Efferocytosis in Maintaining Cardiovascular Homeostasis

**Figure 2:**
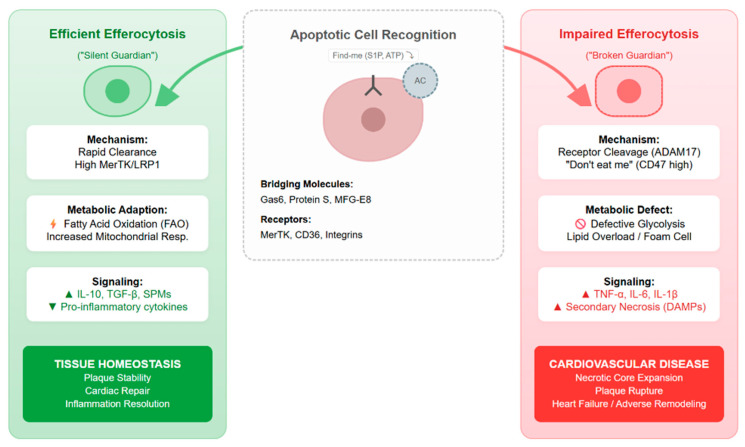
The pivotal role of efferocytosis in determining the balance between tissue homeostasis and cardiovascular pathology. This schematic illustrates the pivotal bifurcation in macrophage fate following apoptotic cell recognition. (**Left**) Under physiological conditions, efficient efferocytosis triggers metabolic adaptation (e.g., fatty acid oxidation) and the secretion of specialized pro-resolving mediators, thereby ensuring inflammation resolution and tissue homeostasis. (**Right**) In disease states, impaired efferocytic capacity—exacerbated by receptor cleavage (e.g., ADAM17) or inhibitory signaling (e.g., CD47)—leads to secondary necrosis and the release of damage-associated molecular patterns (DAMPs). This functional failure perpetuates a pro-inflammatory cycle that drives the progression of atherosclerosis and myocardial infarction.

Efferocytosis is a fundamental physiological process required for the preservation of cardiovascular stability through the regulation of immune responses and cellular metabolism (shown in Figure 2). Under steady-state conditions, the rate of apoptosis in vascular and cardiac tissues is counterbalanced by the efficient clearance of apoptotic cells (ACs) [22]. This process prevents secondary necrosis and the subsequent release of intracellular damage-associated molecular patterns (DAMPs), such as HMGB1 and genomic DNA, which are potent initiators of sterile inflammation [23].

The homeostatic function of efferocytosis is characterized by the induction of immune quiescence. Upon the recognition and internalization of ACs, phagocytes undergo functional reprogramming, leading to the increased synthesis of anti-inflammatory cytokines, such as interleukin-10 (IL-10) and transforming growth factor-beta (TGF-β), and the concurrent inhibition of pro-inflammatory mediators like TNF-α and IL-12 [22,23]. Furthermore, evidence indicates that efferocytosis triggers metabolic adaptation within the phagocyte. The ingestion of AC-derived cargo provides metabolic substrates that enhance fatty acid oxidation (FAO) and mitochondrial oxidative phosphorylation (OXPHOS) [24]. This metabolic shift, driven by the SIRT1-mediated signaling pathway, is essential for sustaining the energetic requirements of continuous phagocytic activity and promotes the maintenance of a pro-repair M2-like macrophage phenotype [25]. Consequently, efferocytosis ensures the structural integrity of the vasculature and myocardium by maintaining a non-inflammatory microenvironment.

## 4. Efferocytosis in Cardiovascular Diseases

### 4.1. Atherosclerosis

Research on efferocytosis and atherosclerosis has garnered much attention in recent years, with defective efferocytosis leading to necrosis and inflammation as an important factor in the formation of atherosclerosis. Atherosclerosis is a major cause of coronary heart disease, cerebral infarction, and peripheral vascular disease, and the lesions are based on impaired lipid metabolism. The lesions begin in the intima, with progressive metamorphosis and calcification of the middle layer of the artery, resulting in thickening and stiffening of the arterial wall and narrowing of the lumen, often involving the large- and medium-sized arteries [26]. In early stages of atherosclerosis, monocyte-derived macrophages are recruited into the atherosclerotic lesion, where they exhibit effective efferocytosis, modulate inflammatory response, and clear the lesion—a process that slows the progression of plaque [27]. However, during the stage of plaque development, macrophages undergo cellular reprogramming that reduces their ability to perform efferocytosis, leading to post-apoptotic necrosis and inflammation of apoptotic cells. As the plaque progresses, efferocytosis may fail, leading to the accumulation of secondary necrotic cells and the formation of a highly inflammatory “necrotic core”—a hallmark of advanced atherosclerosis. This is associated with the type of atherosclerotic plaque that causes heart attacks and strokes [27,28]. In addition, in advanced atherosclerosis, defective efferocytosis is a major driver of necrotic core formation, triggering plaque rupture and acute thrombotic cardiovascular events.

#### 4.1.1. MFG-E8

**Figure 3:**
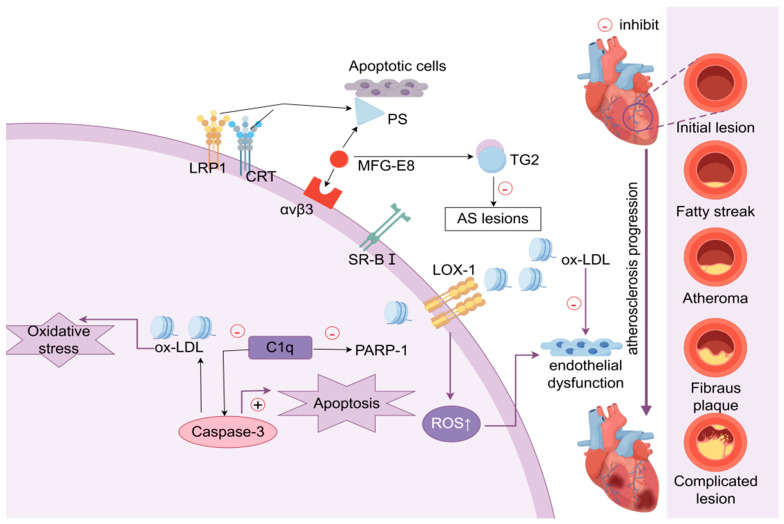
Molecular pathways of efferocytosis in cardiovascular pathology. MFG-E8 binds integrin αvβ3 to PS located on the surface of apoptotic cells, but its expression is reduced in the mid- to late stages of atherosclerosis development. MFGE8 can bind directly to TG2, which is essential for cholesterol transport reversal and the prevention of plaque progression. Furthermore, C1q promotes macrophage viability by reducing the levels of active caspase-3 protein and PARP-1. Additionally, it has the potential to augment phagocytosis through the regulation of oxidized or acetylated LDL, enhance the functionality of cytosolic cells, and impede the progression of atherosclerosis. LRP1, together with its co-receptor CRT, enhances phagocytosis of apoptotic cells by acting on PS.

MFG-E8 acts as a bridging molecule that binds integrin αvβ3 to PS located on the surface of apoptotic cells, and its expression is reduced in the mid-to-late stages of atherosclerosis development. Mice transplanted with MFGE8-deficient bone marrow have an increased necrotic core area at the atherosclerosis plaque lesion and an increased number of apoptotic cells, indicating defective efferocytosis [29]. Notably, MFGE8 can also bind directly to transglutaminase 2 (TG2) [30], which is important for the reversal of cholesterol transport and for stopping plaque progression. C1q is another important bridging molecule, and Ldlr^−/−^ mice lacking C1q develops more atherosclerosis lesions [26]. C1q enhances macrophage viability by downregulating the levels of active caspase-3 protein and polyadenylate diphosphate ribosyltransferase-1 (PARP-1). It can also enhance phagocytosis by recognizing and regulating the modified form of low-density lipoprotein (LDL) (oxidation or acetylation), improve the function of cytosolic cells, and thus delay atherosclerosis [25] (shown in Figure 3).

However, the biological effects of these bridging molecules are highly dependent on the stage of atherosclerotic progression. While C1q and MFG-E8 facilitate the non-inflammatory clearance of apoptotic cells in early-stage lesions [29], their roles may shift in advanced disease. For instance, in advanced atherosclerosis, C1q can activate the classical complement pathway, which potentially enhances leukocyte recruitment and promotes plaque vulnerability [25]. Similarly, while MFG-E8 is essential for apoptotic cell clearance and tissue repair, its physiological function may shift under chronic inflammatory or aging conditions. Recent evidence indicates that MFG-E8 can act as a pro-inflammatory mediator in the aging vasculature, contributing to arterial wall thickening and negative remodeling, which contrasts with its protective role in early injury [31]. These findings underscore the necessity of developing therapeutic strategies that account for the specific disease stage.

#### 4.1.2. Low-Density Lipoprotein Receptor-Related Protein 1

Yancey et al. reported accelerated atherosclerosis plaque formation, increased necrotic core size, and increased residual apoptotic cells in lesions after transplanting bone marrow from LRP1-deficient mice to LDLR^−/−^ mice [32]. Moreover, bone marrow–derived macrophage LRP1 deficiency can cause upregulation of IL-1β, IL-6, and tumor necrosis factor-α (TNF-α) expression and promote vascular inflammation without causing an imbalance in overall lipid levels [33]. It can also lead to significant inhibition of efferocytosis capacity, accumulation of apoptotic macrophages, and enlargement of necrotic core [33]. LRP1 has an important effect on the maintenance of normal efferocytosis, and abnormal LRP1 function may directly contribute to the development of atherosclerosis lesions. In addition, LRP1, together with its co-receptor CRT, enhances phagocytosis of apoptotic cells by acting on PS, which in turn connects to the complement factor C1q [32] (shown in Figure 3). Targeting LRP1 to enhance macrophage phagocytic capacity represents a potential therapeutic strategy.

#### 4.1.3. Scavenger Receptor B I (SR-B I)

SR-B I directly links to PtdSer, leading to phosphorylation of Src (Src kinase family of proteins with tyrosine protein kinase activity) and activation of phosphoinositol 3 kinase (PI3K) and Rac1 and downstream signaling [34], promoting the clearance of apoptotic cells and reducing intraplaque inflammatory response. Specific inhibition of SR-B I expression in macrophages leads to impaired efferocytosis function in both mice and isolated cells and causes upregulation of the expression of inflammatory factors such as IL-6, IL-1β, and TNF-α [35].

#### 4.1.4. Apolipoprotein E (ApoE)

ApoE knockout mice with a tyrosine kinase defect in the MerTK receptor have an increased area of necrotic core at the atherosclerosis plaque lesion and an increased number of apoptotic cells, indicating a defect in efferocytosis function [36]. MerTK deficiency promotes the development of cardiomyopathy [37].

#### 4.1.5. Extracellular Signal-Regulated Kinase 5 (ERK5)

ERK5 regulates the expression of various phagocytic receptors and bridging molecules in macrophages, including MerTK, C1q, and Gas6 [19], which significantly enhances macrophage-mediated efferocytosis. ERK5 inactivation leads to an increase in necrotic cores within plaques. The formation of atherosclerosis lesions in plaques is accelerated and necrotic cores are more unstable when ERK5-specific blocked ApoE−/− mice are fed a high cholesterol diet [37]. Activation of ERK5 may enhance macrophage efferocytic capacity and delay plaque progression.

#### 4.1.6. CD47

The anti-atherosclerosis effects of the integrin-related protein CD47 include the prevention of plaque progression as well as the prevention of plaque rupture and enlargement of the necrotic core. Acting on the extracellular region of the Src homologous structural domain 2 protein tyrosine phosphatase substrate 1 (SHPS-1) of macrophages, the cytoplasmic region of SHPS-1 binds SHP-1 and SHP-2 to form the SHPS-1–SHP-2 complex, which subsequently inhibits cytosolic burial via Syk or PI3K signaling molecules, thereby leading to necrotic core enlargement [38]. Some other molecules, e.g., miRNAs such as miR-126 and miRNA 155, regulate the role of efferocytosis in atherosclerosis [39]. Blockade of CD47 signaling can restore efferocytosis, with CD47-blocking antibodies showing promise in preclinical models [40].

Based on numerous existing studies, we have identified four main causes of impaired efferocytosis during the progression of atherosclerosis: (i) Competitive recognition of phagocytic receptors: the presence of large amounts of reactive oxygen species (ROS) and oxidized low-density lipoprotein (ox-LDL) in atherosclerotic plaques, which competitively bind to phagocytic receptors such as sR-B I or the bridging molecule MFG-E8 [41], affects the recognition process of apoptotic cells. (ii) Attenuation of the “eat me” signal: ox-LDL seems to mask the “eat me” signal of apoptotic cells, which blocks their recognition. (iii) Loss of macrophage-associated phagocytic receptors: during the progression of atherosclerosis, macrophage-related phagocytosis receptors such as CD36, Merk, and LRP1 are lost, resulting in diminished macrophage efferocytosis [42]. (iv) Expression of the “do not eat me” signal: which is usually reduced in apoptotic cells, but this is not the case in atherosclerosis. This may be due to the increased expression of Toll-like receptor 4 (TLR4) during atherosclerosis, which leads to increased expression of pro-inflammatory factors such as TNF-α and IL-1β and decreased expression of anti-inflammatory factors such as TGF-β [43]. TNF-α promotes the expression of CD47, a “do not eat me” signaling molecule on the surface of apoptotic cells, which prevents the recognition and timely clearance of apoptotic cells, leading to increased inflammation and autoimmune reactions [40].

Since the ability of efferocytosis is reduced in advanced atherosclerosis, if drugs that improve efferocytosis can be administrated before the advanced stages of atherosclerosis, the progression of atherosclerosis may be inhibited and the disease may be treated in a better way. A new approach that may successfully target defective efferocytosis is to use antibodies to block CD47—enhancing the function of efferocytosis cells by blocking the protein hydrolysis of the efferocytosis receptor. One study identified a glucocorticoid product, membrane-linked protein A1, that enhanced cellular efferocytosis in mice, relieving inflammation and delaying atherosclerosis [44]. The most beneficial therapy would be to both reduce the “do not eat me” signal in dead cells and maximize efferocytosis capacity [45]. Agonists can be used to induce alternative macrophage development (e.g., PPARγ, PPARδ, LXR) or to promote the conversion of macrophages to an anti-inflammatory pro-catabolic state (e.g., lipocalin, catabolite) while limiting TNF-α production, inflammatory NF-κB activation, and CD47 expression in dead cells. Therapeutic drugs targeting “eat me” signals and related drugs carrying functionalized nanoparticles of miRNA are also currently under development [19,46].

### 4.2. Myocardial Infarction and Heart Failure

**Figure 4:**
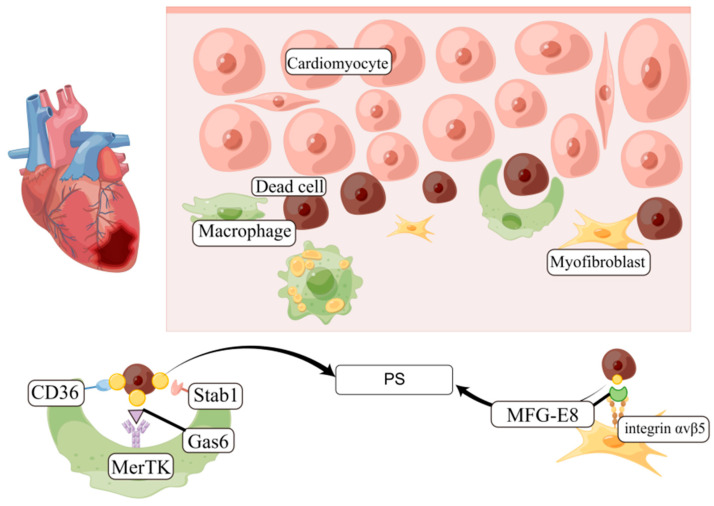
Efferocytosis during myocardial infarction. Efferocytosis through the recognition of PtdSer in the context of an infarcted heart is a crucial process. Within the infarcted heart, various cardiac cells experience cell death, including necrosis and apoptosis. The efferocytic activity of macrophages or myofibroblasts is facilitated by the utilization of either the Gas6/MerTK pathway or the MFG-E8/integrin avb5 pathway.

Worldwide, myocardial infarction is one of the leading causes of death. Efferocytosis plays a key role in the repair of myocardium after myocardial infarction. Thus, if the efferocytosis mechanism is impaired, it can lead to poor remodeling of the heart in the context of chronic inflammation, further resulting in heart failure [47] (shown in Figure 4).

#### 4.2.1. MerTK

Through Gas6 or protein S, MerTK recognizes PS on dead cells [47,48]. Mice lacking macrophage MerTK responded markedly to left anterior descending ischemia–reperfusion injury, as evidenced by AC accumulation, increased infarct size, and reduced cardiac function. The efferocytosis of mouse macrophages was detected using knockout (KO) of MerTK or LRP, a phagocytic receptor. According to Wan et al. [49], MerTK KO mice exhibited reduced phagocytic activity, as opposed to LRP KO mice. In line with these findings, an increase in unphagocytosed apoptotic cells was observed following myocardial infarction in Mertk KO mice compared with their wild-type counterparts, alongside a decrease in cardiomyocytes engulfed by macrophages in Mertk KO mice. It is evident that MerTK contributes to the efferocytosis process of macrophages in the context of myocardial infarction. The extracellular structural domain of MerTK undergoes cleavage by protein 17 (ADAM17), which possesses disintegrin and metalloproteinase properties. Notably, the expression of ADAM17, an exfoliating protease, is upregulated during myocardial infarction [50], resulting in the solubilization of MerTK and its transformation into a soluble Mer [51]. Intriguingly, soluble Mer is found in abundance in the serum of both myocardial infarction patients and model mice [52]. The cleavage of MerTK by ADAM17 results in a decrease in functional MerTK on macrophages and also leads to the competitive inhibition of MerTK by soluble Mers. In mice carrying the MerTKCR mutant, which is resistant to cleavage by ADAM17, the size of the infarct induced by ischemia/reperfusion is reduced, resulting in an enhanced cardiac function. This suggests that inhibiting ADAM17 during myocardial infarction can enhance the clearance of dead cells by macrophages and improve cardiac function.

#### 4.2.2. MFG-E8

It has been shown that myofibroblasts produce MFG-E8, a protein that is secreted and that binds to PS [53], facilitating the engulfment of deceased cardiomyocytes through the interaction with the AVB5 integrin receptor expressed on myofibroblasts. Subsequently, it has been discovered that certain macrophages also secrete MFG-E8. The absence of MFG-E8 in KO mice results in an elevated presence of non-engulfed apoptotic cells following myocardial infarction, along with increased inflammation in the infarcted region. These effects ultimately lead to the deterioration of cardiac function after myocardial infarction [54]. In contrast, the administration of MFG-E8 demonstrates a significant reduction in the quantity of non-phagocytosed apoptotic cells and the level of inflammation within the myocardial infarct region, consequently leading to an enhanced cardiac condition after myocardial infarction. The myofibroblasts exhibit downregulated expression of IL1B, CXCL2, and IL-6 and upregulated expression of TGF-β [54]. While the production of TGF-β by macrophages is typically efficacious, the production of TGF-β by myofibroblasts has the potential to exacerbate hyperfibrosis, thereby posing a potential detrimental effect in this particular context.

#### 4.2.3. CD47

Similarly to atherosclerosis, acute coronary syndrome (ACS) that arises from myocardial injury exhibits abnormally elevated levels of CD47. Administering anti-CD47 antibodies promptly following myocardial infarction enhances the resolution of myocardial inflammation, diminishes the extent of tissue damage, and maintains cardiac function. Several molecules are believed to play a role in the cytokinesis of recruited macrophages [22]. Specifically, CD36 binds to PS, and the absence of CD36 exacerbates cardiac function following myocardial infarction in mice [55]. However, the role of CD36-mediated defective efferocytosis in exacerbating cardiac function after myocardial infarction remains unclear, as CD36 is a multifunctional protein. A study by Wan et al. showed that macrophages isolated from CD36 KO mice did not exhibit decreased phagocytic activity, indicating that further analysis is needed to determine whether macrophages phagocytose via CD36 during myocardial infarction [49]. Additionally, Stab1, a receptor that directly recognizes PS, has been reported to be upregulated in macrophages during myocardial infarction [56]. However, it is unclear whether Stab1 contributes to cytokinesis during myocardial infarction.

#### 4.2.4. Unresolved Inflammation Plays a Crucial Role in the Progression of Advanced Heart Failure

In cases of ACS and myocardial infarction, the initial acute inflammation caused by plaque rupture initiates a subsequent response that can result in prolonged cardiac damage. When a severe and prolonged inflammatory response persists, it leads to adverse remodeling, and ultimately to heart failure [57]. Loxs plays a crucial role in the regulation of hydroxylation of polyunsaturated fatty acids (PUFAs) and the synthesis of lipid mediators that possess both pro- and anti-inflammatory effects [58]. Lipids facilitate the elimination of deceased or apoptotic neutrophils through efferocytosis. The absence of 12/15LOX in mice results in effective catabolism and cardiac healing, interpreted as a compensatory elevation in anti-inflammatory epoxyeicosatrienoic acids (EETs) via the cytochrome P450 (CYP) pathway, thereby contributing to the restoration of cardiac function [59]. MaResin is synthesized through the 12-LOX pathway from DHA [60]. It exhibits a diverse range of effects, such as diminishing neutrophil accumulation following injury, promoting phagocytosis and efferocytosis of apoptotic cells, expediting tissue regeneration at the injury site, and inducing a shift in macrophage phenotype towards an anti-inflammatory/pro-ablative state. Consequently, MaResin serves as a potent modulator of both the local inflammatory response and the stem cell activity.

#### 4.2.5. Distinctions in Efferocytic Requirements Between Atherosclerosis and Myocardial Infarction

The requirements for efferocytosis in myocardial infarction (MI) differ significantly from those in atherosclerosis in terms of kinetics and scale. Atherosclerosis involves a chronic, progressive accumulation of apoptotic cells within the arterial wall, where macrophages exhibit a gradual exhaustion of phagocytic capacity due to persistent lipid overload and chronic oxidative stress [61]. Conversely, MI represents an acute event requiring the rapid clearance of millions of apoptotic and necrotic cardiomyocytes to mitigate the localized cytokine storm and facilitate the formation of a stable fibrotic scar [49]. In the post-MI microenvironment, the primary objective of efferocytosis is to prevent cardiac rupture by ensuring the structural replacement of dead cells with collagen-rich tissue, whereas in atherosclerosis, the focus remains on preventing the conversion of a stable fibroatheroma into a vulnerable plaque [26].

### 4.3. Other Cardiovascular Diseases

In addition to ischemic heart disease such as myocardial infarction and heart failure, other cardiovascular ailments are also linked to chronic inflammation, for which the mechanisms of degradation have been comprehensively elucidated.

#### 4.3.1. Myocarditis

During the acute phase of myocarditis, a significant quantity of IL-17a is present, which facilitates the shedding of MERTK and influences the expression profile of genes associated with inflammation and tissue remodeling. Previous studies have demonstrated that cardiac myocytes are capable of inducing the shedding of macrophage MERTK as a way to inhibit phagocytosis [62]. This piece of finding underscores an alternative mechanism through which IL-17a triggers the shedding of MERTK via cardiac fibroblasts, thereby contributing to the cardiac pathology observed in autoimmune myocarditis. Additionally, Hou et al. discovered a significant reduction in levels of myeloid MERTK among human myocarditis patients in comparison with ischemic patients [63]. Inhibition of IL-17a or prevention of MERTK shedding may restore efferocytic capacity and mitigate disease progression.

#### 4.3.2. Cardiometabolic Diseases

The maintenance of metabolic homeostasis is intricately linked to the efficient clearance of apoptotic cells. Defective efferocytosis has emerged as a critical pathophysiological mechanism underlying the spectrum of cardiometabolic diseases, which intertwines obesity, insulin resistance, diabetes, and their cardiovascular complications. Accumulating evidence indicates that obesity and insulin resistance create a systemic environment detrimental to efferocytosis. Macrophages derived from leptin-deficient (ob/ob) mice or from dietary-induced obese models exhibit significantly impaired phagocytic capacity towards apoptotic cells [64,65]. Specifically, Ldlr^−/−/ob/ob^ mice exhibit compromised efferocytosis and larger necrotic nuclei in the context of atherosclerosis, compared with Ldlr^−/−^ mice. However, when Ldlr^−/−/ob/ob^ mice were administered with a diet abundant in ω-3 fatty acids, their macrophages demonstrated an enhanced rate of efferocytosis, although the underlying mechanisms remain incompletely understood [65]. Insulin resistance itself can directly impair efferocytic signaling. The PI3K/Akt pathway, a crucial downstream effector of insulin signaling, is also involved in the cytoskeletal rearrangements necessary for phagocytosis. Therefore, systemic insulin resistance may lead to “cellular blindness” in macrophages, hindering their ability to respond to ‘eat-me’ signals and contributing to the accumulation of apoptotic debris in metabolic tissues [66]. This discovery may provide insight into the susceptibility of obesity as well as offer diabetic individuals with complications related to resolution defects, including impaired wound healing and atherosclerosis. Targeting the PI3K/Akt pathway, supplementing with ω-3 fatty acids, or modulating CD36 function represent potential strategies to improve efferocytosis in metabolic syndrome.

## 5. Discussion

Efferocytosis functions as a central mechanism for maintaining tissue homeostasis by linking cell death with immune resolution and tissue repair. The impairment of this process in cardiovascular diseases leads to the accumulation of apoptotic cells and secondary necrosis, which drives chronic inflammation. While the signaling pathways responsible for the recognition and engulfment of dying cells are well established, the dysfunction of efferocytosis is context-dependent. The mechanisms of failure vary significantly between the chronic progression of atherosclerosis and the acute onset of myocardial infarction.

A primary challenge in developing therapeutic strategies is that bridging molecules exhibit distinct and often opposing effects depending on the disease stage. Studies indicate that proteins such as MFG-E8 and C1q function differently in early versus advanced pathology. MFG-E8 facilitates the clearance of apoptotic cells in early vascular injury, but in the aging vasculature, it promotes arterial wall thickening and negative remodeling [31]. Similarly, C1q supports the clearance of modified lipids and dead cells during the initial stages of atherosclerosis; however, in advanced plaques, it activates the complement pathway, which exacerbates inflammation and promotes plaque instability [25]. This stage-specific variability implies that systemically increasing the levels of these molecules could produce detrimental effects in advanced disease states. Therefore, therapeutic interventions requires precise modulation based on the pathological stage rather than broad upregulation.

The mechanisms required to restore efferocytosis also differ between atherosclerosis and myocardial infarction. In atherosclerosis, the primary obstacle is the chronic reduction in phagocytic capacity within lipid-rich plaques. Exposure to oxidized lipids and reactive oxygen species impairs receptor function and phagolysosomal fusion. In this chronic setting, blocking the CD47 signaling pathway effectively inhibits the negative regulation of phagocytosis, thereby reactivating macrophages and limiting the expansion of the necrotic core [40]. In contrast, myocardial infarction involves an acute loss of functional receptors due to the rapid inflammatory response. The enzyme ADAM17 cleaves the MerTK receptor from the macrophage surface, which significantly reduces the capacity to clear dying cardiomyocytes [52]. Consequently, strategies that inhibit ADAM17-mediated shedding are more relevant for acute ischemia than strategies targeting CD47. This distinction demonstrates that therapeutic approaches must be tailored to the specific kinetic requirements of the cardiovascular condition.

In addition to receptor signaling, systemic metabolic conditions directly influence the efficiency of efferocytosis. Diabetes and insulin resistance impair the ability of macrophages to reorganize their cytoskeleton for engulfment, even when surface receptors are intact. This functional impairment involves defects in the PI3K/Akt signaling pathway and the dysfunction of scavenger receptors such as CD36 [66]. This observation suggests that in patients with metabolic syndrome, restoring efferocytosis requires addressing the underlying metabolic deficits of the phagocytes. As a result, agents that modulate cellular metabolism, such as LXR agonists, may be necessary to support the energy-demanding process of clearance [25].

Finally, the clinical translation of these findings requires methods to improve specificity and safety. Systemic inhibition of CD47 poses a risk of anemia due to the clearance of erythrocytes. To address this, biomimetic nanoparticles have been developed to deliver gene-silencing agents specifically to plaque macrophages, thereby minimizing systemic toxicity [46]. For acute injury, the use of specialized pro-resolving mediators, such as maresins, can inhibit ADAM17 activation and maintain MerTK integrity [60]. These findings collectively suggest that effective therapeutic strategies must synchronize receptor availability with the metabolic competence of phagocytes and utilize targeted delivery systems to minimize off-target effects.

## 6. Conclusions

Efferocytosis is an indispensable mechanism for the elimination of apoptotic cells in the body. It protects the tissue microenvironment from harmful effects of catabolic cells, without which secondary necrosis of apoptotic cells can occur, leading to a range of inflammatory and other injuries. Furthermore, efferocytosis also opens new avenues for the treatment of cardiovascular-related diseases. Improving the function of efferocytosis provides novel insights into the direction of therapy, as cardiovascular diseases can be treated by facilitating or blocking steps involved in the efferocytosis process. The therapeutic potential of efferocytosis in the field of cardiovascular diseases should be comprehensively explored in the near future.

## Figures and Tables

**Table 1:** Signaling molecules related to efferocytosis.

Phase	Molecule (Apoptotic Cell)	Bridging Molecule	Recognition Receptor (Phagocytic Cell)
Recruitment	CX3CL1	-	CX3CR1
	ICAM3	-	CD14
	LPC	-	G2A
	S1P	-	S1PRs
	ATP, UTP	-	P2Y2
Recognition	PtdSer	-	BAI-1/TIM-1/3/4/stabilin-2/RAGE
	Calr	-	LRP1
Tethering and signaling	PtdSer	Gas6	TAM (TYRO3, AXL, MerTK)
	PtdSer	PROS	TAM (TYRO3, AXL, MerTK)
	PtdSer	MFGE8	αvβ3/αvβ5
	PtdSer	C1q	SCARF1/Integrin αMβ2
Phagocytosis	p130cas–CrkII–Dock180 complex		

CX3CL1, CX3C chemokine ligand 1; CX3CR1, CX3C chemokine receptor 1; ICAM3, intercellular adhesion molecule 3; LPC, lysophosphatidylcholine; G2A, G-protein–coupled receptor; S1P, sphingosine-1-phosphate; S1PRs, sphingosine-1-phosphate receptors; ATP/UTP, adenosine triphosphate/uridine triphosphate; P2Y2, purinergic receptor P2Y2; PtdSer, phosphatidylserine; BAI-1, brain-specific angiogenesis inhibitor 1; TIM, T-cell immunoglobulin- and mucin-domain–containing molecule; RAGE, receptor for advanced glycation end products; Calr, calreticulin; LRP1, low-density lipoprotein receptor-related protein 1; Gas6, growth arrest-specific gene 6 product; AXL, Anexelekto receptor tyrosine kinase; MerTK, Mer tyrosine kinase; MFGE8, milk fat globule–EGF factor 8; C1q, complement 1q; SCARF1, scavenger receptor F1.

## Data Availability

No new data were created or analyzed in this study. Data sharing is not applicable to this article.

## References

[1] Elliott M.R., Ravichandran K.S. (2016). The Dynamics of Apoptotic Cell Clearance. Dev. Cell.

[2] Gheibi Hayat S.M., Bianconi V., Pirro M., Sahebkar A. (2019). Efferocytosis: Molecular mechanisms and pathophysiological perspectives. Immunol. Cell Biol..

[3] Parnaik R., Raff M.C., Scholes J. (2000). Differences between the clearance of apoptotic cells by professional and non-professional phagocytes. Curr. Biol..

[4] Allen J.E., Rückerl D. (2017). The Silent Undertakers: Macrophages Programmed for Efferocytosis. Immunity.

[5] Kumar S., Birge R.B. (2016). Efferocytosis. Curr. Biol..

[6] Henson P.M., Bratton D.L., Fadok V.A. (2001). Apoptotic cell removal. Curr. Biol..

[7] Lemke G. (2019). How macrophages deal with death. Nat. Rev. Immunol..

[8] Elliott M.R., Chekeni F.B., Trampont P.C., Lazarowski E.R., Kadl A., Walk S.F., Park D., Woodson R.I., Ostankovich M., Sharma P. (2009). Nucleotides released by apoptotic cells act as a find-me signal to promote phagocytic clearance. Nature.

[9] Nagata S. (2018). Apoptosis and Clearance of Apoptotic Cells. Annu. Rev. Immunol..

[10] Birge R.B., Boeltz S., Kumar S., Carlson J., Wanderley J., Calianese D., Barcinski M., Brekken R.A., Huang X., Hutchins J.T. (2016). Phosphatidylserine is a global immunosuppressive signal in efferocytosis, infectious disease, and cancer. Cell Death Differ..

[11] Kawano M., Nagata S. (2018). Efferocytosis and autoimmune disease. Int. Immunol..

[12] Thornley T.B., Fang Z., Balasubramanian S., Larocca R.A., Gong W., Gupta S., Csizmadia E., Degauque N., Kim B.S., Koulmanda M. (2014). Fragile TIM-4-expressing tissue resident macrophages are migratory and immunoregulatory. J. Clin. Investig..

[13] Lian S., Xie R., Ye Y., Xie X., Li S., Lu Y., Li B., Cheng Y., Katanaev V.L., Jia L. (2019). Simultaneous blocking of CD47 and PD-L1 increases innate and adaptive cancer immune responses and cytokine release. EBioMedicine.

[14] Wang L., Li H., Tang Y., Yao P. (2020). Potential Mechanisms and Effects of Efferocytosis in Atherosclerosis. Front. Endocrinol..

[15] Martinez J. (2017). Prix Fixe: Efferocytosis as a Four-Course Meal. Curr. Top. Microbiol. Immunol..

[16] Tzelepis F., Verway M., Daoud J., Gillard J., Hassani-Ardakani K., Dunn J., Downey J., Gentile M.E., Jaworska J., Sanchez A.M.J. (2015). Annexin1 regulates DC efferocytosis and cross-presentation during Mycobacterium tuberculosis infection. J. Clin. Investig..

[17] Akakura S., Singh S., Spataro M., Akakura R., Kim J.I., Albert M.L., Birge R.B. (2004). The opsonin MFG-E8 is a ligand for the alphavbeta5 integrin and triggers DOCK180-dependent Rac1 activation for the phagocytosis of apoptotic cells. Exp. Cell Res..

[18] Albert M.L., Kim J.I., Birge R.B. (2000). alphavbeta5 integrin recruits the CrkII-Dock180-rac1 complex for phagocytosis of apoptotic cells. Nat. Cell Biol..

[19] Tajbakhsh A., Rezaee M., Kovanen P.T., Sahebkar A. (2018). Efferocytosis in atherosclerotic lesions: Malfunctioning regulatory pathways and control mechanisms. Pharmacol. Ther..

[20] Maimon N., Zamir Z.Z., Kalkar P., Zeytuni-Timor O., Schif-Zuck S., Larisch S., Ariel A. (2020). The pro-apoptotic ARTS protein induces neutrophil apoptosis, efferocytosis, and macrophage reprogramming to promote resolution of inflammation. Apoptosis Int. J. Program. Cell Death.

[21] Schutters K., Kusters D.H.M., Chatrou M.L.L., Montero-Melendez T., Donners M., Deckers N.M., Krysko D.V., Vandenabeele P., Perretti M., Schurgers L.J. (2013). Cell surface-expressed phosphatidylserine as therapeutic target to enhance phagocytosis of apoptotic cells. Cell Death Differ..

[22] Doran A.C., Yurdagul A., Tabas I. (2020). Efferocytosis in health and disease. Nat. Rev. Immunol..

[23] Zhang B., Zou Y., Yuan Z., Jiang K., Zhang Z., Chen S., Zhou X., Wu Q., Zhang X. (2024). Efferocytosis: The resolution of inflammation in cardiovascular and cerebrovascular disease. Front. Immunol..

[24] Zhang S., Weinberg S., DeBerge M., Gainullina A., Schipma M., Kinchen J.M., Ben-Sahra I., Gius D.R., Yvan-Charvet L., Chandel N.S. (2019). Efferocytosis Fuels Requirements of Fatty Acid Oxidation and the Electron Transport Chain to Polarize Macrophages for Tissue Repair. Cell Metab..

[25] Thielens N.M., Tedesco F., Bohlson S.S., Gaboriaud C., Tenner A.J. (2017). C1q: A fresh look upon an old molecule. Mol. Immunol..

[26] Yurdagul A.J., Doran A.C., Cai B., Fredman G., Tabas I.A. (2017). Mechanisms and Consequences of Defective Efferocytosis in Atherosclerosis. Front. Cardiovasc. Med..

[27] Yahagi K., Kolodgie F.D., Otsuka F., Finn A.V., Davis H.R., Joner M., Virmani R. (2016). Pathophysiology of native coronary, vein graft, and in-stent atherosclerosis. Nat. Rev. Cardiol..

[28] Hansson G.K., Libby P., Tabas I. (2015). Inflammation and plaque vulnerability. J. Intern. Med..

[29] Ait-Oufella H., Kinugawa K., Zoll J., Simon T., Boddaert J., Heeneman S., Blanc-Brude O., Barateau V., Potteaux S., Merval R. (2007). Lactadherin deficiency leads to apoptotic cell accumulation and accelerated atherosclerosis in mice. Circulation.

[30] Boisvert W.A., Rose D.M., Boullier A., Quehenberger O., Sydlaske A., Johnson K.A., Curtiss L.K., Terkeltaub R. (2006). Leukocyte transglutaminase 2 expression limits atherosclerotic lesion size. Arterioscler. Thromb. Vasc. Biol..

[31] Chiang H.Y., Chu P.H., Lee T.H. (2019). MFG-E8 mediates arterial aging by promoting the proinflammatory phenotype of vascular smooth muscle cells. J. Biomed. Sci..

[32] Yancey P.G., Ding Y., Fan D., Blakemore J.L., Zhang Y., Ding L., Zhang J., Linton M.F., Fazio S. (2011). Low-density lipoprotein receptor-related protein 1 prevents early atherosclerosis by limiting lesional apoptosis and inflammatory Ly-6Chigh monocytosis: Evidence that the effects are not apolipoprotein E dependent. Circulation.

[33] Yancey P.G., Blakemore J., Ding L., Fan D., Overton C.D., Zhang Y., Linton M.F., Fazio S. (2010). Macrophage LRP-1 controls plaque cellularity by regulating efferocytosis and Akt activation. Arterioscler. Thromb. Vasc. Biol..

[34] Tao H., Yancey P.G., Babaev V.R., Blakemore J.L., Zhang Y., Ding L., Fazio S., Linton M.F. (2015). Macrophage SR-BI mediates efferocytosis via Src/PI3K/Rac1 signaling and reduces atherosclerotic lesion necrosis. J. Lipid Res..

[35] Yu P., Qian A.S., Chathely K.M., Trigatti B.L. (2018). PDZK1 in leukocytes protects against cellular apoptosis and necrotic core development in atherosclerotic plaques in high fat diet fed ldl receptor deficient mice. Atherosclerosis.

[36] Thorp E., Cui D., Schrijvers D.M., Kuriakose G., Tabas I. (2008). Mertk receptor mutation reduces efferocytosis efficiency and promotes apoptotic cell accumulation and plaque necrosis in atherosclerotic lesions of apoe^−/−^ mice. Arterioscler. Thromb. Vasc. Biol..

[37] Giurisato E., Xu Q., Lonardi S., Telfer B., Russo I., Pearson A., Finegan K.G., Wang W., Wang J., Gray N.S. (2018). Myeloid ERK5 deficiency suppresses tumor growth by blocking protumor macrophage polarization via STAT3 inhibition. Proc. Natl. Acad. Sci. USA.

[38] Russ A., Hua A.B., Montfort W.R., Rahman B., Riaz I.B., Khalid M.U., Carew J.S., Nawrocki S.T., Persky D., Anwer F. (2018). Blocking “don’t eat me” signal of CD47-SIRPα in hematological malignancies, an in-depth review. Blood Rev..

[39] Suresh Babu S., Thandavarayan R.A., Joladarashi D., Jeyabal P., Krishnamurthy S., Bhimaraj A., Youker K.A., Krishnamurthy P. (2016). MicroRNA-126 overexpression rescues diabetes-induced impairment in efferocytosis of apoptotic cardiomyocytes. Sci. Rep..

[40] Kojima Y., Volkmer J.P., McKenna K., Civelek M., Lusis A.J., Miller C.L., Direnzo D., Nanda V., Ye J., Connolly A.J. (2016). CD47-blocking antibodies restore phagocytosis and prevent atherosclerosis. Nature.

[41] Linton M.F., Babaev V.R., Huang J., Linton E.F., Tao H., Yancey P.G. (2016). Macrophage Apoptosis and Efferocytosis in the Pathogenesis of Atherosclerosis. Circ. J. Off. J. Jpn. Circ. Soc..

[42] Gonzalez L., Trigatti B.L. (2017). Macrophage Apoptosis and Necrotic Core Development in Atherosclerosis: A Rapidly Advancing Field with Clinical Relevance to Imaging and Therapy. Can. J. Cardiol..

[43] Costales P., Castellano J., Revuelta-López E., Cal R., Aledo R., Llampayas O., Nasarre L., Juarez C., Badimon L., Llorente-Cortés V. (2013). Lipopolysaccharide downregulates CD91/low-density lipoprotein receptor-related protein 1 expression through SREBP-1 overexpression in human macrophages. Atherosclerosis.

[44] Sugimoto M.A., Ribeiro A.L.C., Costa B.R.C., Vago J.P., Lima K.M., Carneiro F.S., Ortiz M.M.O., Lima G.L.N., Carmo A.A.F., Rocha R.M. (2017). Plasmin and plasminogen induce macrophage reprogramming and regulate key steps of inflammation resolution via annexin A1. Blood.

[45] Tajbakhsh A., Gheibi Hayat S.M., Butler A.E., Sahebkar A. (2019). Effect of soluble cleavage products of important receptors/ligands on efferocytosis: Their role in inflammatory, autoimmune and cardiovascular disease. Ageing Res. Rev..

[46] Tajbakhsh A., Bianconi V., Pirro M., Gheibi Hayat S.M., Johnston T.P., Sahebkar A. (2019). Efferocytosis and Atherosclerosis: Regulation of Phagocyte Function by MicroRNAs. Trends Endocrinol. Metab. TEM.

[47] DeBerge M., Zhang S., Glinton K., Grigoryeva L., Hussein I., Vorovich E., Ho K., Luo X., Thorp E.B. (2017). Efferocytosis and Outside-In Signaling by Cardiac Phagocytes. Links to Repair, Cellular Programming, and Intercellular Crosstalk in Heart. Front. Immunol..

[48] Lemke G. (2017). Phosphatidylserine Is the Signal for TAM Receptors and Their Ligands. Trends Biochem. Sci..

[49] Wan E., Yeap X.Y., Dehn S., Terry R., Novak M., Zhang S., Iwata S., Han X., Homma S., Drosatos K. (2013). Enhanced efferocytosis of apoptotic cardiomyocytes through myeloid-epithelial-reproductive tyrosine kinase links acute inflammation resolution to cardiac repair after infarction. Circ. Res..

[50] Zheng D.Y., Zhao J., Yang J.M., Wang M., Zhang X.T. (2016). Enhanced ADAM17 expression is associated with cardiac remodeling in rats with acute myocardial infarction. Life Sci..

[51] Thorp E., Vaisar T., Subramanian M., Mautner L., Blobel C., Tabas I. (2011). Shedding of the Mer tyrosine kinase receptor is mediated by ADAM17 protein through a pathway involving reactive oxygen species, protein kinase Cδ, and p38 mitogen-activated protein kinase (MAPK). J. Biol. Chem..

[52] DeBerge M., Yeap X.Y., Dehn S., Zhang S., Grigoryeva L., Misener S., Procissi D., Zhou X., Lee D.C., Muller W.A. (2017). MerTK Cleavage on Resident Cardiac Macrophages Compromises Repair After Myocardial Ischemia Reperfusion Injury. Circ. Res..

[53] Hanayama R., Tanaka M., Miwa K., Shinohara A., Iwamatsu A., Nagata S. (2002). Identification of a factor that links apoptotic cells to phagocytes. Nature.

[54] Nakaya M., Watari K., Tajima M., Nakaya T., Matsuda S., Ohara H., Nishihara H., Yamaguchi H., Hashimoto A., Nishida M. (2017). Carcdiac myofibroblast engulfment of dead cells facilitates recovery after myocardial infarction. J. Clin. Investig..

[55] Dehn S., Thorp E.B. (2018). Myeloid receptor CD36 is required for early phagocytosis of myocardial infarcts and induction of Nr4a1-dependent mechanisms of cardiac repair. FASEB J. Off. Publ. Fed. Am. Soc. Exp. Biol..

[56] Ryabov V., Gombozhapova A., Rogovskaya Y., Kzhyshkowska J., Rebenkova M., Karpov R. (2018). Cardiac CD68+ and stabilin-1+ macrophages in wound healing following myocardial infarction: From experiment to clinic. Immunobiology.

[57] Halade G.V., Lee D.H. (2022). Inflammation and resolution signaling in cardiac repair and heart failure. EBioMedicine.

[58] Ackermann J.A., Hofheinz K., Zaiss M.M., Krönke G. (2017). The double-edged role of 12/15-lipoxygenase during inflammation and immunity. Biochim. Biophys. Acta Mol. Cell Biol. Lipids.

[59] Kain V., Ingle K.A., Rajasekaran N.S., Halade G.V. (2021). Activation of EP4 receptor limits transition of acute to chronic heart failure in lipoxygenase deficient mice. Theranostics.

[60] Jadapalli J.K., Halade G.V. (2018). Unified nexus of macrophages and maresins in cardiac reparative mechanisms. FASEB J. Off. Publ. Fed. Am. Soc. Exp. Biol..

[61] Tabas I. (2009). Macrophage apoptosis in atherosclerosis: Consequences on plaque progression and the role of endoplasmic reticulum stress. Antioxid. Redox Signal..

[62] Zhang S., Yeap X.Y., Grigoryeva L., Dehn S., DeBerge M., Tye M., Rostlund E., Schrijvers D., Zhang Z.J., Sumagin R. (2015). Cardiomyocytes induce macrophage receptor shedding to suppress phagocytosis. J. Mol. Cell. Cardiol..

[63] Hou X., Chen G., Bracamonte-Baran W., Choi H.S., Diny N.L., Sung J., Hughes D., Won T., Wood M.K., Talor M.V. (2019). The Cardiac Microenvironment Instructs Divergent Monocyte Fates and Functions in Myocarditis. Cell Rep..

[64] Luo B., Wang Z., Zhang Z., Shen Z., Zhang Z. (2019). The deficiency of macrophage erythropoietin signaling contributes to delayed acute inflammation resolution in diet-induced obese mice. Biochim. Biophys. Acta Mol. Basis Dis..

[65] Li S., Sun Y., Liang C.P., Thorp E.B., Han S., Jehle A.W., Saraswathi V., Pridgen B., Kanter J.E., Li R. (2009). Defective phagocytosis of apoptotic cells by macrophages in atherosclerotic lesions of ob/ob mice and reversal by a fish oil diet. Circ. Res..

[66] Byrne C.D. (2013). Ectopic fat, insulin resistance and non-alcoholic fatty liver disease. Proc. Nutr. Soc..

